# Complete genome and functional insights into *Microbacterium* sp. strain FBCC-B4120, a novel freshwater isolate with diverse biotechnological traits

**DOI:** 10.1371/journal.pone.0347549

**Published:** 2026-05-07

**Authors:** Jina Kim, Hyaekang Kim, Jaeduk Goh, Chun-Zhi Jin, Hyung-Gwan Lee, Chang Soo Lee, Sanghwa Park, Eu Jin Chung, Yejin Park, Seungmin Shin, Youngmin Han, Woori Kwak

**Affiliations:** 1 Department of Biotechnology, The Catholic University of Korea, Bucheon, Republic of Korea; 2 Department of Agricultural Biotechnology and Research Institute of Agriculture and Life Sciences, Seoul National University, Seoul, Republic of Korea; 3 Bio-resources Bank Division, Nakdonggang National Institute of Biological Resources, Sangju, Republic of Korea; 4 Prokaryote Research Division, Biological Resources Research Department, Nakdonggang National Institute of Biological Resources, Sangju, Republic of Korea; 5 Cell Factory Research Center, Korea Research Institute of Bioscience and Biotechnology (KRIBB), Daejeon, Republic of KoreaKorea; 6 Fungi Research Division, Biological Resources Research Department, Nakdonggang National Institute of Biological Resources, Sangju, Republic of Korea; 7 Division of Cardiology, Emory University School of Medicine, Atlanta, Georgia, United States of America; 8 Department of Medical and Biological Sciences, The Catholic University of Korea, Bucheon, Republic of Korea; Duy Tan University: Dai Hoc Duy Tan, VIET NAM

## Abstract

The genus *Microbacterium* encompasses a diverse group of Actinobacteria with broad ecological distribution and functional versatility. Here, we report the complete genome sequence of *Microbacterium* sp. FBCC-B4120, a novel strain isolated from a freshwater ecosystem in Korea. The genome was assembled into a single circular chromosome of 3.78 Mb with a G + C content of 70.5%, achieving 100% genome completeness. Phylogenomic analysis confirmed that FBCC-B4120 represents a distinct species within the genus, with *M. thalassium* identified as its closest relative. FBCC-B4120 demonstrated diverse physiological activities such as antimicrobial activity, siderophore production, glycosidase activity, and traits associated with anti-obesity and antiviral effects. Comparative genome analysis revealed a broad repertoire of carbohydrate-active enzymes and a distinct urea utilization cluster, suggesting expanded capacities for carbohydrate metabolism and nitrogen utilization. These findings establish FBCC-B4120 as a novel *Microbacterium* species with distinctive genomic features and multifunctional traits, highlighting its potential as a valuable resource for future biotechnological applications. However, the functional predictions presented here rely primarily on genomic annotation, and further biochemical validation will be required to confirm these activities.

## Introduction

The genus *Microbacterium*, belonging to the phylum Actinobacteria, was first established by Orla-Jensen in 1919 [1]. This genus is Gram-positive, aerobic, non-motile rods that produce carotenoid pigments, typically resulting in a yellowish colony appearance [2]. To date, more than 160 validly published species have been described (https://lpsn.dsmz.de/genus/microbacterium), and the genus continues to expand through the discovery of novel species. *Microbacterium* species have been isolated from a variety of environments, including soils, marine habitats, plant rhizospheres, extreme environments, and even clinical specimens, underscoring their remarkable ecological adaptability [3–8].

The growing discovery of *Microbacterium* strains with diverse metabolic and ecological functions has highlighted their importance across industrial and environmental fields. Several strains have been studied as plant growth-promoting bacteria (PGPB), heavy metal bioremediators, and producers of industrial enzymes. For instance, *M. testaceum* exhibits notable plant growth-promoting traits such as indole-3-acetic acid production, siderophore synthesis, and biocontrol activity, making it a potential agricultural bioresource [9]. Other isolates from contaminated environments have demonstrated resistance and bioaccumulation capacity toward cadmium, cobalt, nickel, and chromium, suggesting possible applications in heavy metal bioremediation [10]. In the context of industrial biotechnology, the esterase EstSIT01 from *M. chocolatum* SIT101 has been identified as highly suitable for the synthesis of chiral intermediates in d-biotin production, demonstrating its utility as a biocatalyst [11]. Some *Microbacterium* strains have been reported to contribute to ecosystem processes, including organic matter cycling and pollutant degradation. *M. lacus*, isolated from lake sediments, was found to degrade the agricultural antibiotic sulfadiazine, suggesting its potential role in mitigating pharmaceutical contamination in aquatic systems [12]. In addition, *M. fluvii*, obtained from submerged wood in river ecosystems, has been shown to metabolize diverse carbon sources and produce extracellular enzymes, indicating its potential contribution to organic matter decomposition in freshwater environments [13].

In this study, we report the complete genome assembly of *Microbacterium* sp. FBCC-B4120, a novel strain from a freshwater ecosystem, and conduct comparative genome analysis with other *Microbacterium* species. In addition to genome-based phylogenetic characterization, we evaluated the physiological activities of FBCC-B4120, including antimicrobial activity, β-glucosidases activity, siderophore synthesis, and potential anti-obesity and antiviral properties. By integrating genomic and phenotypic findings, this study aims to elucidate the biological features and potential applications of FBCC-B4120, thereby expanding our understanding of the functional diversity within the genus *Microbacterium*.

## Materials and methods

### Strain information

Soil samples were collected from the brackish water zone of Suncheon Bay, Korea (34°53′4.21″N, 127°30′42.16″E), serially diluted, and cultured on R2A agar. Distinct colonies were purified, and one isolate was designated *Microbacterium* sp. FBCC-B4120. Isolated strain is banked and maintained by the Freshwater Bioresources Culture Collection (FBCC), Nakdonggang National Institute of Biological Resources (NNIBR), Republic of Korea. The strain FBCC-B4120 was obtained from the FBCC-B4120 and used in this study.

### Ethics statement

This study did not involve any field sampling. The strain analyzed was obtained from the Freshwater Bioresources Culture Collection (FBCC), Nakdonggang National Institute of Biological Resources (NNIBR), Republic of Korea (catalog/accession: FBCC-B4120). The FBCC conducts original collection and curation in compliance with applicable regulations and institutional standard procedures (ISO 9001:2015). Therefore, no field access or collection permits were required for the work reported here.

### Assessment of functional activities

Representative plate images of the functional assays are provided in Fig S1 in S1 File, and the corresponding quantitative measurements (n = 3) are summarized in Table S3 in S1 File.

### Antibacterial activity assays

Antimicrobial activity was evaluated using a two-layer agar diffusion assay. The culture supernatant of strain FBCC-B4120 was filtered through a 0.22-μm membrane and applied (150 μL) to an 8-mm paper disc. Each disc was placed on R2A agar containing 5% of an *Escherichia coli* ATCC 25922 suspension (OD₆₀₀ = 0.5). Plates were incubated at 30 °C for 48 h, and inhibition zones (mm) were measured. For comparison, R2A broth was used as a negative control. Inhibition zones for FBCC-B4120 and streptomycin controls were measured in triplicate.

### Siderophore production and phosphate solubilization

Siderophore production was assessed on Chrome Azurol S (CAS) agar (orange halo formation) [14], and phosphate solubilization on National Botanical Research Institute’s Phosphate (NBRIP) agar (clear halo formation) [15].

### β-glucosidase activity assays

β-glucosidase activity was assessed using two complementary approaches. Esculin Iron Agar was employed, where dark halos around colonies indicated enzymatic activity and were measured in millimeters. Chromogenic polysaccharide substrates from the AZCL series (e.g., AZCL-amylose, Megazyme, Wicklow, Ireland) were used according to the manufacturer’s instructions.

### Pancreatic lipase and α-glucosidase inhibition assays

Porcine pancreatic lipase, p-nitrophenyl butyrate (p-NPB), and Orlistat (all from Sigma-Aldrich) were used for the lipase inhibition assay. The reaction mixture was prepared by incubating the lipase solution with ethyl acetate extract of cell culture, followed by addition of p-NPB and subsequent incubation at 37 °C with shaking. Absorbance at 410nm was measured using a microplate reader (TECAN, Spark). The inhibitory activity was calculated using according to the following equation:


$$Lipase\;inhibitions\;(\%)=\left(\text{1}-\frac{A-A^{\prime }}{B-B^{\prime }}\right)\times \text{1}\text{0}\text{0}$$

Where all parameters represent absorbance at 410 nm; A corresponds to the reaction mixture containing both the sample and p-NPB, A′ to the sample blank (without p-NPB), B to the control containing p-NPB only, and B′ to the control blank (without p-NPB). Antiviral potential was evaluated by measuring α-glucosidase inhibition using p-nitrophenyl-α-D-glucopyranoside as the substrate, as previously described [16].

### DNA extraction and genome sequencing

Genomic DNA of high quality was isolated with the Mag-Bind^®^ Universal Pathogen Kit (Omega Bio-Tek, Norcross, GA, USA) following the supplier’s instructions. DNA integrity and concentration were verified using both a NanoDrop 2000 spectrophotometer (Thermo Fisher Scientific, Waltham, MA, USA) and a Qubit 4 fluorometer equipped with the dsDNA High Sensitivity Assay Kit (Thermo Fisher Scientific, Waltham, MA, USA). For long-read sequencing, libraries were generated using the ligation sequencing kit (SQK-LSK114, Oxford Nanopore Technologies, Oxford, UK) and run on a Flongle R10.4 flow cell (FLO-FLG114, ONT). For short-read sequencing, libraries were prepared with the Illumina TruSeq Nano DNA kit and processed on the Illumina NovaSeq X platform (Illumina, San Diego, CA, USA)

### Genome assembly

Nanopore basecalling was conducted using Guppy v6.5.7 [17] with the super accuracy model. Trimming for adapter sequence in the generated reads was conducted using Porechop_ABI v0.5.0 [18]. Trimmed reads were assembled using Flye v2.9.2 [19] with nano-hq parameter. For a more accurate genome sequence, hybrid assembly was conducted using long reads and short reads. Medaka v1.8.0 (https://github.com/nanoporetech/medaka, accessed in May 2023) was used for initial polishing with a super-accuracy calling model. The further polishing process was conducted using Pilon v.1.24 [20] for hybrid assembly. Short reads were mapped to the assembled genome using Bowtie2 v2.5.1 [21] with --no-mixed option (only proper pair read mapping) for more reliable read mapping, and the generated bam file was used as input for the Pilon polishing. Genome assembly completeness was evaluated using Benchmarking Universal Single-Copy Orthologs (BUSCO) v5.7.1 [22] with micrococcales_odb10. A Tetra Correlation Search was conducted using JSpeciesWS [23].

### Comparative genome analysis

To analyze the evolutionary relationship of FBCC-B4120, comparative analyses were performed against 41 publicly available *Microbacterium* genomes: 40 complete genomes of other *Microbacterium* species and the contig-level assembly of *M. thalassium* obtained from NCBI Genome database (https://www.ncbi.nlm.nih.gov/datasets/genome/, accessed in May 2023). Prokka v1.14.6 [24] and Proksee (https://proksee.ca/) were used for gene annotation and construction of a circular genome map. The species delineation thresholds were analyzed using PYANI v0.2.13.1 [25] and the GGDC web server [26]. Average nucleotide identity (ANI) values were obtained using the BLAST method, while digital DNA-DNA hybridization (dDDH) values were calculated using formula 2. Pan-genome analysis was performed using Roary v3.13.0 [27] with the parameters -i 80 and -cd 100 to obtain aligned core gene sequences. For 16S rRNA genes, representative sequences from each genome were extracted and aligned using MAFFT v7.526 [28]. The aligned sequences of both core genes and 16S rRNA genes were trimmed with Gblocks v9.1 [29] to remove ambiguously aligned regions. Maximum likelihood phylogenetic trees were constructed using IQ-TREE 2 v2.4.0 [30] with the best-fit substitution model and ultrafast bootstrap approximation with 1,000 replicates. Clusters of Orthologous Genes (COG) functional annotation was performed using eggNOG-mapper v2.1.13 [31]. Carbohydrate-active enzymes were identified using the dbCAN v5.1.2 [32] against the Carbohydrate-Active enZymes database (CAZy) (https://www.cazy.org/). KEGG Orthology assignments and pathway reconstruction were carried out using KEGG Koala [33]. The functional association networks were constructed using STRING web server [34]. The complete proteome of FBCC-B4120, predicted from the annotated genome, was uploaded to STRING, and individual genes of interest were queried to generate interaction networks. Networks were constructed with default parameters and a medium confidence threshold (score ≥0.4) and visualized in Cytoscape v3.10.3 [35].

## Results and discussion

### Whole-genome features and phylogenomic placement of microbacterium sp. FBCC-B4120

**Fig 1:**
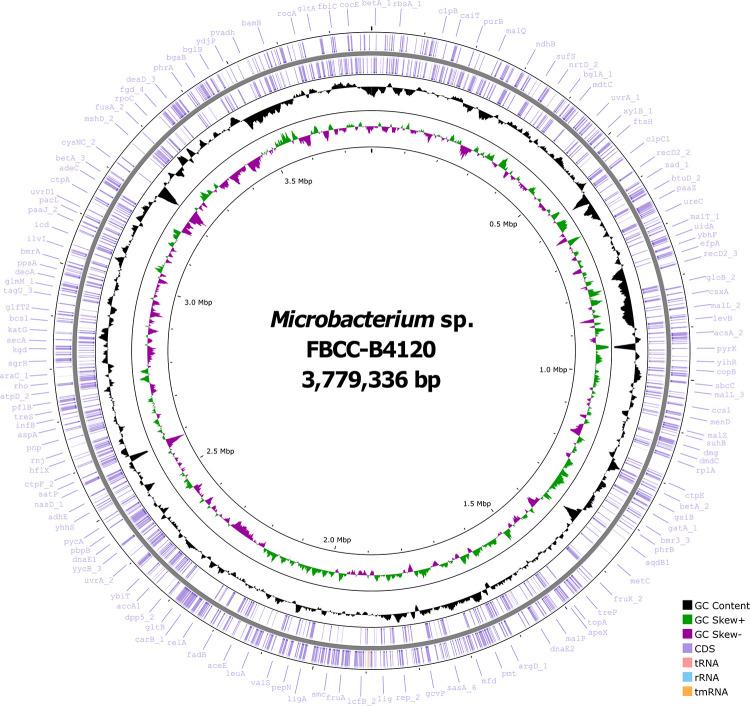
Circular genome map of *Microbacterium sp.* FBCC-B4120. The complete genome consists of a single circular chromosome (3,779,336 bp) with a G + C content of 70.5%. From the outer to the inner circle: predicted coding sequences (CDSs), tRNA, rRNA, and tmRNA genes are indicated, followed by GC content and GC skew. Representative functional genes are labeled on the outer track.

In this study, a complete and high-quality genome of *Microbacterium* sp. FBCC-B4120 was assembled using a hybrid approach combining Oxford Nanopore long-read and Illumina short-read sequencing data (Fig 1). Genome completeness, as evaluated by BUSCO analysis, was 100% (537 complete BUSCOs: 532 single-copy and 5 duplicated). The genome was assembled into a single circular chromosome without plasmids, totaling 3.78 Mb with a G + C content of 70.5%. Gene annotation identified 3,437 coding sequences, 6 rRNA genes, 54 tRNA genes, and 1 tmRNA (Table 1). Taxonomic affiliation was examined using Tetra Correlation Search (TCS) in JSpeciesWS, which confirmed assignment to the genus *Microbacterium* and revealed the highest similarity (Z-score > 0.997) with *M. thalassium*.

**Table 1:** Whole genome sequence overview of FBCC-B4120.

Species Name	*Microbacterium* sp. FBCC-B4120
NCBI Taxonomy ID	33882
Domain	Bacteria
Taxonomy	Bacteria; Terrabacteria group; Actinomycetota; Actinomycetes; Micrococcales; Microbacteriaceae
Genome Size (bp)	3,779,336
GC content in the DNA	70.5 mol% G + C
Number of Genome Sequences	1 Circular (Single chromosomal DNA without plasmid)
Number of Plasmids	0
Number of Coding Sequences	3,437
Number of rRNAs	6
Number of tRNAs (tmRNA)	54 (1)

**Fig 2:**
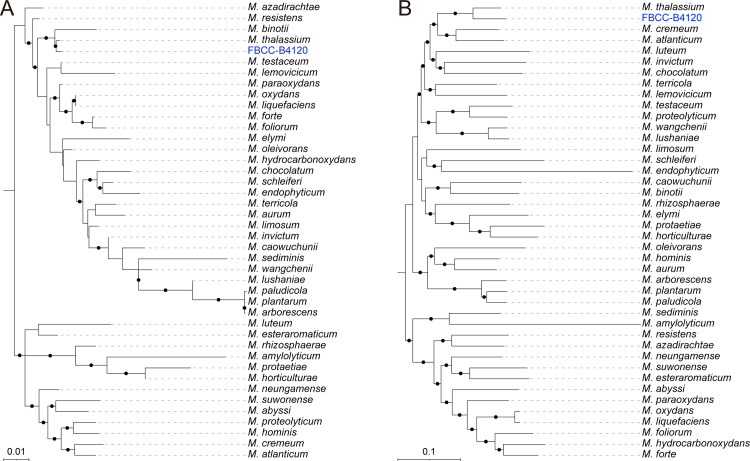
Phylogenomic analysis of *Microbacterium sp.* FBCC-B4120. FBCC-B4120 is highlighted in blue. Both phylogenies consistently place FBCC-B4120 as a sister lineage to M. thalassium, with strong bootstrap support. (A) Maximum-likelihood tree based on the 16S rRNA gene sequence. (B) Maximum-likelihood tree based on the core gene set from pan-genome analysis.

To further resolve the phylogenetic position of FBCC-B4120, we compared it with 40 publicly available complete genomes from the genus and the reference genome of *M. thalassium* (S1 Table in S1 File). ANI and dDDH values, which are standard criteria for prokaryotic species delineation (ANI ≥ 95–96% and dDDH ≥ 70%) [36], showed that FBCC-B4120 shared ANI of 85.6% (alignment coverage 67.3%) and dDDH of 30.1% with *M. thalassium* (S2 Table in S1 File). These values are well below the accepted thresholds, indicating that FBCC-B4120 represents a phylogenetically related but distinct species within the genus *Microbacterium*. Phylogenomic trees were reconstructed using both the 16S rRNA gene sequence and the core gene set derived from pan-genome analysis. In the 16S rRNA gene-based tree, FBCC-B4120 clustered with *M. thalassium* with strong bootstrap support (98%), forming a sister lineage characterized by a short branch length (Fig 2A). Together with *M. binotii*, they formed a clade adjacent to the *M. azadirachtae*–*M. resistens* group. Consistently, the core gene-based tree also placed FBCC-B4120 as a sister lineage to *M. thalassium*, with 100% bootstrap support (Fig 2B). In contrast to the 16S rRNA tree, however, the branch length separating FBCC-B4120 and *M. thalassium* was relatively longer, highlighting clearer genome-wide divergence.

These results indicate that FBCC-B4120 represents a novel species within the genus *Microbacterium*, consistently forming a sister lineage to *M. thalassium*. Interestingly, both species were isolated from saline-associated environments, with FBCC-B4120 obtained from brackish soil and *M. thalassium* from the mangrove rhizosphere [37]. While this ecological correspondence suggests that adaptation to elevated salinity, further genomic and functional evidence would be required to substantiate this link.

### Functional and physiological characterization of FBCC-B4120

To evaluate the industrial potential of FBCC-B4120, we assessed its antimicrobial activity, plant growth-promoting traits, extracellular enzyme production, and physiological enzyme activities (S1 Figure and S3 Table in S1 File).

FBCC-B4120 formed a clear inhibition zone of 24.77 ± 0.54 mm (n = 3) against *Escherichia coli* ATCC 25922, indicating antibacterial activity.

Siderophore production was confirmed by the CAS assay, which formed an orange halo of 5.17 ± 0.29 mm. Siderophores are high-affinity iron-chelating metabolites secreted under iron-limiting conditions and known to play important roles in rhizosphere colonization and microbial competition [38]. By contrast, phosphate solubilization was not detected, suggesting that FBCC-B4120 contributes to iron acquisition but lacks phosphate-solubilizing capacity.

β-glucosidase activity assays on Esculin Iron Agar revealed a dark zone of 21.33 ± 1.15 mm, indicating strong enzymatic activity. β-glucosidase, a subclass of glycosidases, specifically cleave β-glycosidic bonds in carbohydrates, releasing glucose from diverse β-glucosides including disaccharides, oligosaccharides, and plant-derived glycosides [39,40]. These enzymes play essential roles in cellulose and biomass degradation, carbon cycling, and microbial nutrient acquisition, and they are of considerable industrial importance for biofuel production, food processing, and the synthesis or modification of pharmaceutical glycosides [41–44].

Physiological enzyme assays further revealed a pancreatic lipase inhibition rate of 24.38 ± 0.68% and an α-glucosidase inhibition rate of 41.22 ± 16.79%. Pancreatic lipase is a key enzyme in triglyceride hydrolysis and a major therapeutic target for anti-obesity drug development [45,46], whereas α-glucosidase is implicated in glycoprotein processing in host and viral systems [47,48]. These inhibitory effects suggest potential relevance of FBCC-B4120 to anti-obesity and antiviral mechanisms, although further validation is required.

Collectively, FBCC-B4120 exhibits multiple functional traits, including *E. coli*-specific antimicrobial activity, siderophore production, strong β-glucosidase activity, and bioactive enzyme inhibition. These findings demonstrate its potential industrial relevance. However, the precise metabolites and genetic determinants underlying these activities remain to be clarified. Further genome-guided analyses of metabolic pathways and biosynthetic gene clusters will be required to elucidate the mechanisms and to evaluate the application potential of this strain.

### Comparative genomics insights into the functional repertoire of FBCC-B4120

To further characterize the functional genomic landscape of FBCC-B4120, comparative pan-genome and functional annotation analyses were conducted using 41 publicly available *Microbacterium* genomes. The pan-genome comprised 76,156 genes, including 195 core genes shared by all strains and 75,961 accessory genes. FBCC-B4120 contributed 1,052 unique genes.

**Fig 3:**
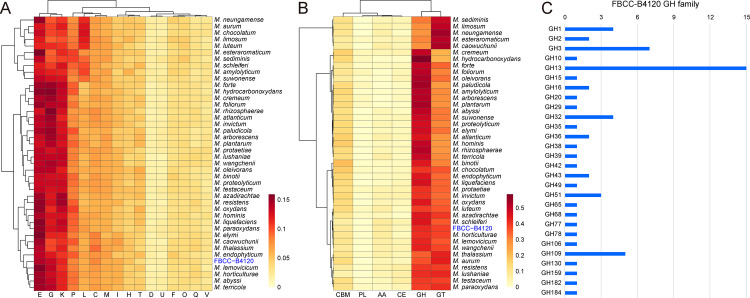
Functional genome profiling of *Microbacterium sp*. FBCC-B4120 in the context of the genus. (A) Heatmap of row-normalized proportions for selected COG functional categories across 41 *Microbacterium* genomes. Categories shown: E, amino acid transport & metabolism; G, carbohydrate transport & metabolism; K, transcription; P, inorganic ion transport & metabolism; L, replication, recombination & repair; C, energy production & conversion; M, cell wall/membrane/envelope biogenesis; I, lipid transport & metabolism; H, coenzyme transport & metabolism; T, signal transduction mechanisms; D, cell cycle control, division & chromosome partitioning; U, intracellular trafficking, secretion & vesicular transport; F, nucleotide transport & metabolism; O, posttranslational modification, protein turnover & chaperones; Q, secondary metabolite biosynthesis, transport & catabolism; V, defense mechanisms. Higher color intensity indicates a greater within-genome proportion for that category. (B) Heatmap of row-normalized proportions of CAZy top-level classes (GH, GT, PL, CE, CBM, and AA) across *Microbacterium* genomes. (C) Distribution of glycoside hydrolase (GH) families in strain FBCC-B4120, highlighting the most abundant GH families.

Across the genus, the dominant COG categories were E (amino acid transport & metabolism), G (carbohydrate transport & metabolism), and K (transcription), which reflect core metabolic and regulatory functions. FBCC-B4120 displayed a relatively higher proportion of category C (energy production & conversion), encompassing genes associated with electron transport chains, ATP synthesis, and respiratory metabolism (Fig 3A). This enrichment suggests enhanced energetic adaptability. Carbohydrate-active enzyme annotations showed that glycoside hydrolases (GHs) and glycosyltransferases (GTs) are the dominant enzyme classes across the genus *Microbacterium*, and FBCC-B4120 exhibited relatively elevated proportions of both families (Fig 3B). GHs are primarily involved in the degradation and conversion of carbohydrates, consistent with the strong β-glucosidase activity observed in this strain. In contrast, GTs are associated with polysaccharide biosynthesis, cell wall formation, and host interactions [49,50]. In FBCC-B4120, 27 GH families were identified. The most abundant were GH13 (15 genes), GH3 (7 genes), GH1 (4 genes), GH32 (4 genes), and GH109 (5 genes) (Fig 3C). Additional families reflect a broad enzymatic repertoire. Among the identified GH families, GH1 and GH3 included several genes annotated as β-glucosidases (Table 2). These β-glucosidase genes are likely to underlie the strong activity observed on Esculin Iron Agar, suggesting that FBCC-B4120 harbors multiple isoenzymes contributing to its robust carbohydrate-degrading capacity.

**Table 2:** β-glucosidase genes of FBCC-B4120 belonging to GH1 and GH3 families.

Gene ID	KO	EC	Preferred name	Description	CAZy	dbCAN
OENLEIBI_00015	K05349	3.2.1.21	*bglX*	Glycosyl hydrolase family 3 (GH3)	GH3	GH3_e128
OENLEIBI_00602	K05349	3.2.1.21	–	GH3 family, β-glucosidase	GH3	GH3_e94
OENLEIBI_03195	K05349	3.2.1.21	–	GH3 family, fibronectin type III-like domain	GH3	GH3_e197
OENLEIBI_03350	K05349	3.2.1.21	*bglK*	GH3 family, fibronectin type III-like domain	GH3	GH3_e154
OENLEIBI_00386	K05350	3.2.1.21	–	Glycosyl hydrolase family 1 (GH1)	–	GH1_e113
OENLEIBI_00769	K05350	3.2.1.21	–	Glycosyl hydrolase family 1 (GH1)	–	GH1_e69
OENLEIBI_02481	K05350	3.2.1.21	–	Glycosyl hydrolase family 1 (GH1)	–	GH1_e113

**Fig 4:**
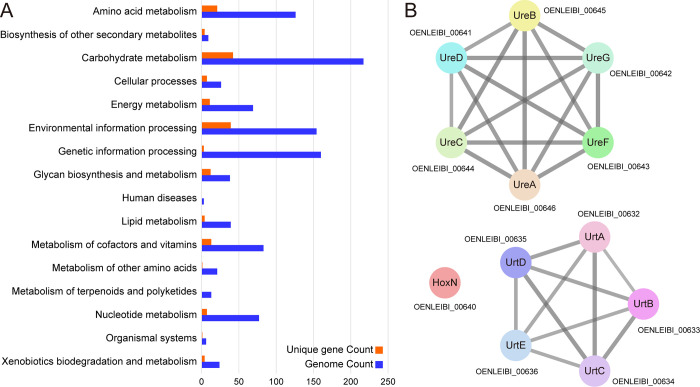
KEGG functional profiling and urea utilization cluster of *Microbacterium* sp. FBCC-B4120. (A) KEGG functional categorization of genome-wide annotated genes and unique genes in FBCC-B4120, illustrating the distribution of metabolic and cellular pathways. (B) STRING-based network representation of the urea utilization cluster, comprising urea transport components (UrtABCDE), nickel transporter (HoxN), and urease structural and accessory proteins (UreABCDFG). The network was reconstructed using a high-confidence cutoff of 0.7, with active sources from Experiments, Databases, Neighborhood, and Gene Fusion.

KEGG functional profiling was conducted to assess the metabolic and functional characteristics of FBCC-B4120 (Fig 4A). The majority of genes were assigned to core metabolic pathways such as Carbohydrate metabolism (217 genes), Genetic information processing (160 genes), Environmental information processing (154 genes), and Amino acid metabolism (126 genes). Among the unique genes, Carbohydrate metabolism (42 genes) and Environmental information processing (39 genes) were most prominent, suggesting potential specialization of the strain in the utilization of diverse carbohydrate resources and adaptation to environmental changes. In addition, a considerable number of unique genes were identified in Amino acid metabolism (21 genes), Metabolism of cofactors and vitamins (13 genes), and Glycan biosynthesis and metabolism (12 genes), suggesting that FBCC-B4120 may harbor genomic traits contributing to nitrogen utilization, cofactor biosynthesis, and glycan metabolism. Interestingly, among the ABC transporter-related unique genes, FBCC-B4120 harbors a complete urea transport system. This observation indicates that a fully assembled Urt operon is present, which allows this strain to utilize urea as a nitrogen source [51].

Gene annotation analysis further identified a gene cluster that allows sequential urea uptake and hydrolysis. A complete set of ABC-type urea transporters (UrtABCDE) was identified. Although Prokka annotated some components as branched-chain amino acid ABC transporters, both eggNOG and KEGG Koala consistently mapped them to the Urt operon, with domain structure (Peripla_BP/ABC_tran/BPD_transp) further supporting this classification (S4 Table in S1 File). Upstream of the operon, TetR family transcriptional regulators were detected, suggesting possible transcriptional regulation of the cluster [52]. Downstream of the Urt operon, modules for urease activation and nickel acquisition were consecutively organized. A high-affinity nickel transporter (HoxN) was present, supplying Ni² ⁺ required for urease activation [53], followed by accessory proteins UreD, UreF, and UreG, and the catalytic subunits UreA, UreB, and UreC [54]. Although a canonical UreE homolog was not detected within the cluster, previous studies in actinobacteria have suggested that UreG/CobW-like proteins may function as alternative metal chaperones [55], which could also be the case here. Taken together, this urea utilization cluster provides a coherent pathway consisting of urea transport (UrtABCDE), nickel uptake (HoxN), and urease assembly and catalysis (UreABCDFG) (Fig 4B). These features suggest that FBCC-B4120 is capable of utilizing urea as a nitrogen source, with urease activity generating ammonia and thereby increasing local pH, which may confer ecological advantages [56,57].

Overall, the comparative genomic analyses indicate that FBCC-B4120 harbors an enriched repertoire of carbohydrate-active enzymes together with a strain-specific urea utilization cluster, providing genomic insights into its multifunctional traits and underscoring its ecological versatility and potential for biotechnological applications. Nevertheless, these insights are derived from *in silico* annotations and comparative analyses; thus, functional validation through targeted biochemical and physiological assays will be necessary to substantiate the predicted roles of these gene clusters.

## Conclusion

In this study, we presented the complete genome sequence of *Microbacterium* sp. FBCC-B4120, a novel freshwater isolate that represents a distinct species within the genus. The high-quality circular genome of FBCC-B4120 (3.78 Mb, 70.5% G + C) showed 100% completeness. Comparative genome metrics further revealed that FBCC-B4120 possesses a genome size and GC content similar to *M. thalassium* but shares only 85.6% ANI and 30.1% dDDH, confirming its species-level divergence.

Phenotypic characterization showed that FBCC-B4120 possesses multiple functional traits, including antimicrobial activity against *E. coli*, siderophore production, glycosidase activity, and additional physiological properties associated with anti-obesity and antiviral effects. Genome-based analyses further complemented these findings by revealing metabolic features that may support ecological adaptation and potential utility. In particular, the presence of a complete urea utilization cluster suggests a coordinated system for urea transport, nickel acquisition, and urease-mediated hydrolysis, which may contribute to nitrogen utilization and persistence in freshwater environments.

Overall, these findings expand current knowledge of the genus *Microbacterium* by introducing novel species with distinct experimental and genomic features. Nevertheless, this study has limitations, as the bioactive compounds underlying the observed physiological activities were not chemically identified, and most functional predictions relied on genomic annotation. Further biochemical and ecological investigations will be essential to clarify the molecular mechanisms and confirm the functional capacities of this strain.

## Supporting information

S1 File S1 Table. List of Mi*crobacterium* species genomes used for comparative and phylogenomic analyses. **S2 Table.** Pairwise ANI and dDDH values between Microbacterium sp. FBCC-B4120 and reference Microbacterium genomes. **S3 Table.** Results of repeated measurements for each activity experiment of Microbacterium sp. FBCC-B4120. **S4 Table.** Predicted genes comprising the urea utilization cluster in Microbacterium sp. FBCC-B4120. **S1 Fig**. Functional assays of Microbacterium sp. FBCC-B4120. (A) R2A broth negative control and streptomycin positive controls for the antimicrobial activity assay against *Escherichia coli* ATCC 25922: (−), R2A broth; 100, streptomycin 100 ppm; 1000, streptomycin 1,000 ppm. (B) Antimicrobial activity against *E. coli* ATCC 25922. (C) Siderophore production on CAS agar. (D) β-glucosidase activity on Esculin Iron Agar.
(ZIP)
