# Supplementary figures and images for: Complete genome and functional insights into Microbacterium sp. strain FBCC-B4120, a novel freshwater isolate with diverse biotechnological traits
Source: PLoS One. 2026 May 7;21(5):e0347549. doi: 10.1371/journal.pone.0347549 (PMC13152184; doi:10.1371/journal.pone.0347549)

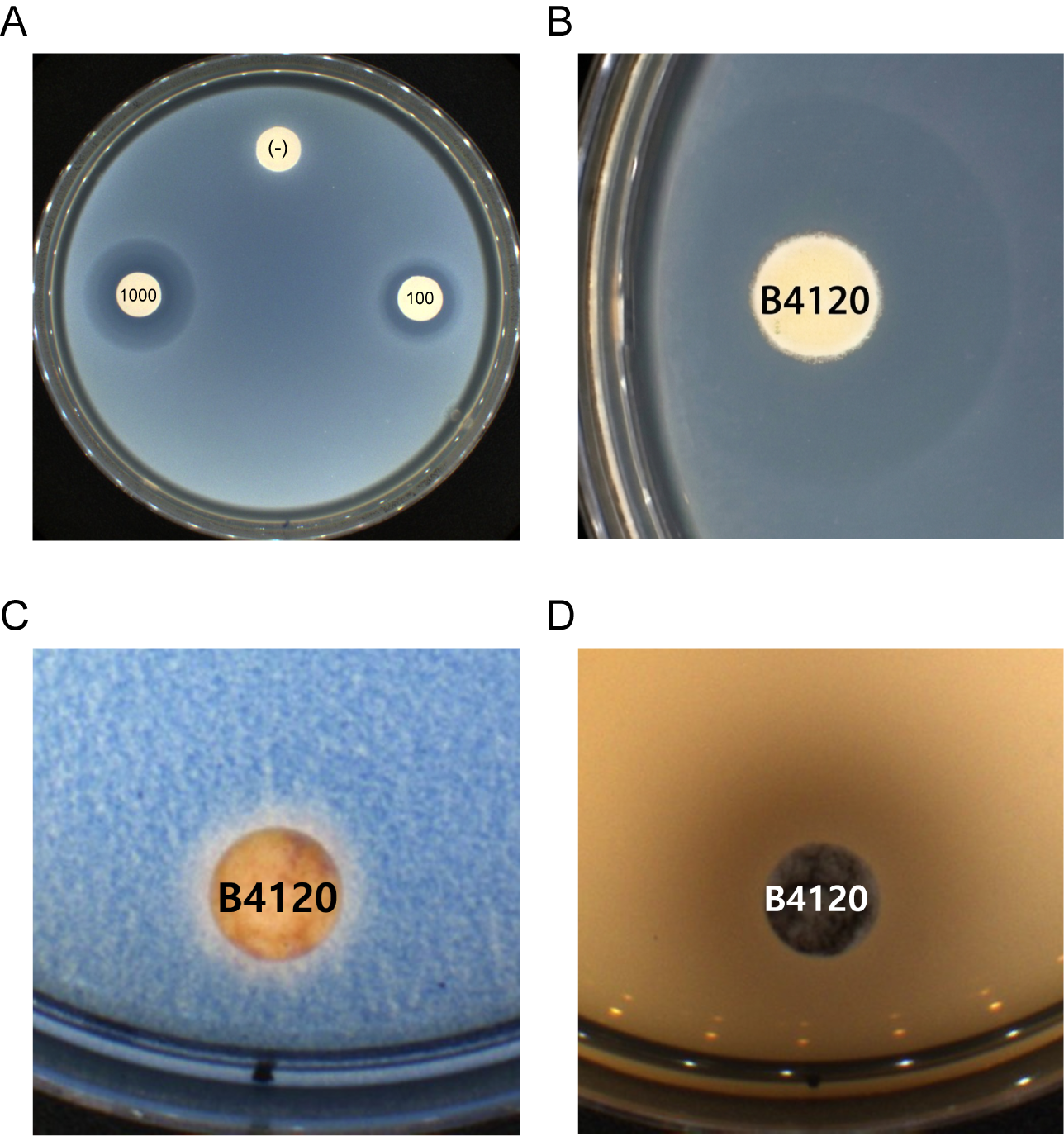

Supplement: S1 File — List of Microbacterium species genomes used for comparative and phylogenomic analyses. S2 Table. Pairwise ANI and dDDH values between Microbacterium sp. FBCC-B4120 and reference Microbacterium genomes. S3 Table. Results of repeated measurements for each activity experiment of Microbacterium sp. FBCC-B4120. S4 Table. Predicted genes comprising the urea utilization cluster in Microbacterium sp. FBCC-B4120. S1 Fig. Functional assays of Microbacterium sp. FBCC-B4120. (A) R2A broth negative control and streptomycin positive controls for the antimicrobial activity assay against Escherichia coli ATCC 25922: (−), R2A broth; 100, streptomycin 100 ppm; 1000, streptomycin 1,000 ppm. (B) Antimicrobial activity against E. coli ATCC 25922. (C) Siderophore production on CAS agar. (D) β-glucosidase activity on Esculin Iron Agar. (ZIP) [file pone.0347549.s001.zip › Supporting_Information/Supplementary_Figure_1.tif]
